# Repurposing Niclosamide as a Therapeutic Drug against Acute Liver Failure by Suppressing Ferroptosis

**DOI:** 10.3390/pharmaceutics15071950

**Published:** 2023-07-14

**Authors:** Xiao Zhong, Xue-Gong Fan, Ruochan Chen

**Affiliations:** 1 Department of Infectious Diseases, Hunan Key Laboratory of Viral Hepatitis, Xiangya Hospital, Central South University, Changsha 410008, China; 22022192@csu.edu.cn (X.Z.); xgfan@csu.edu.cn (X.-G.F.); 2 National Clinical Research Center for Geriatric Disorders, Xiangya Hospital, Central South University, Changsha 410008, China

**Keywords:** ALF, ferroptosis, DEGs, hub genes, niclosamide, LPS/D-GalN, therapeutic, STAT3

## Abstract

Acute liver failure (ALF) is a severe liver disease with a high mortality rate without effective therapeutic drugs. Ferroptosis is a form of programmed cell death that plays an important role in ALF. In this study, we aimed to identify ferroptosis-related genes in ALF, thereby predicting promising compounds to treat ALF. First, mRNA microarray data were utilized to identify the ferroptosis-related differentially expressed genes (DEGs). Hub genes were screened in the protein–protein interaction network and validated. Subsequently, potential drugs to treat ALF were predicted. One of the predicted drugs was tested in an ALF model of mice. Ferroptosis examination and molecular docking were analyzed to explore the mechanism. A total of 37 DEGs were identified, ten hub genes were extracted, and their expression in ALF was validated. The predicted drug niclosamide mitigated lipopolysaccharide/D-galactosamine-induced hepatotoxicity, and decreased mortality of mice in the ALF model. Mechanically, niclosamide may combine with signal transducer and activator of transcription 3 to inhibit ALF progression by suppressing ferroptosis. This study may help advance our understanding of the role of ferroptosis in ALF, and niclosamide may be promising for therapeutic efficacy in patients with ALF.

## 1. Introduction

Acute liver failure (ALF) is a severe liver disease caused by various insults [1]. The chief culprits of ALF in developed countries are acetaminophen-induced hepatotoxicity, ischemia, and other drug-induced liver injuries, whereas viral hepatitis, especially that caused by hepatitis B, accounts for most cases in developing countries [2,3]. ALF has a high mortality rate of up to 30% [1,4]. Currently, the most effective therapy is liver transplantation. However, liver transplantation is characterized by a shortage of liver donors, high expense, and postoperative complications, such as infections and graft rejection in the post-surgery period [5]. Therefore, searching for specific therapeutic drugs for ALF is of great significance.

Ferroptosis has been extensively discussed in liver and non-liver diseases as a gradual iron-controlled, programmed cell death pattern [6]. Unlike other forms of cell death, ferroptosis exhibits specific biological and morphological characteristics. Ferroptosis is characterized by iron metabolism disorder, redox imbalance, and accumulation of lipid peroxidation products [7]. Specific morphological features, especially the change of mitochondrial morphology, present in ferroptosis, including increased mitochondrial membrane density, absence or reduction of mitochondrial cristae, and outer membrane rupture [8]. Ferroptosis can be inhibited by iron chelators (e.g., desferrioxamine mesylate) and lipid peroxidation inhibitors (e.g., vitamin E, liproxstatin, and ferrostatin) [9,10]. The differential expression of several genes were considered a hallmark of ferroptosis, e.g., prostaglandin-endoperoxide synthase 2 (PTGS2/COX2) and acyl-CoA synthetase long-chain family member 4 (ACSL4) [11]. 

Hepatic oxidative stress and inflammation are the key factors leading to ALF. Reactive oxygen species (ROS) and reactive nitrogen species (RNS) were also engaged in ferroptosis [12]. Hepatic ferroptosis is thought to initiate inflammation in cases of nonalcoholic steatohepatitis [13]. Liproxtatin-1 (lip-1) significantly reduced the secretion of inflammatory factors such as TNFα, IL-6, and IL-10 in pulmonary fibrosis by activating the Nrf2/HO-1 signaling axis [14]. Ferroptosis mediates ALF induced by APAP or lipopolysaccharide (LPS)/D-galactosamine (D-GalN) [15,16]. Antagonizing ferroptosis by specific inhibitors or iron chelators could protect mice from hepatotoxicity and improve survival in experimental animals [17,18]. Though ferroptosis plays an important role in ALF, the potential molecular mechanism remains obscure. Current ferroptosis inhibitors have problems such as short half-life, and long research time, which are difficult to translate into clinical applications [19]. Searching for new therapeutic agents, especially old drugs, against ferroptosis remains a big challenge.

The liver plays a crucial role in maintaining immune tolerance to self-antigens while effectively responding to pathogenic challenges [20]. However, dysregulation of immune responses can lead to liver diseases, including autoimmune liver diseases and liver injury [21]. Antigen-presenting cells (APCs), particularly macrophages, assume a pivotal role in the immune response and tissue repair processes in ALF [22]. Macrophages act as key mediators of inflammation and immune regulation within the liver microenvironment [23]. In recent years, the role of ferroptosis in the immune response of liver diseases has received increasing attention from researchers. Indoleamine 2,3-dioxygenase 1 (IDO1) exacerbates ferroptosis in hepatocytes during acute immune hepatitis, particularly in the presence of elevated nitrative stress [24]. Despite the liver’s inherent tolerogenic nature, immune tolerance can be lost due to dysregulation of immunological regulatory mechanisms. This dysregulation allows for uncontrolled immune activation, leading to liver inflammation and injury [25].

Our study, for the first time, aimed to identify the key ferroptosis-related genes and their regulatory network in ALF. In addition, potential drugs targeting ferroptosis were predicted. Most importantly, the traditional oral anthelmintic drug niclosamide was proved to protect mice against ALF by inhibiting ferroptosis, probably through combining with signal transducer and activator of transcription 3 (STAT3). The current study will provide novel insights into the underlying mechanisms associated with the development ALF, thus helping to discover novel potential targets and strategies for ALF diagnosis and treatment. 

## 2. Materials and Methods

### 2.1. Microarray Data

GEO is a public functional tool for users to download array/sequence-based data and achieve gene expression profiles [26]. Raw data of gene expression and detailed information were downloaded from the GEO database. In the current dataset of the GEO database, only four searchable datasets of gene expression profiling were obtained from human liver subjects. Three ALF-correlated GEO datasets (GSE14668, GSE96851 and GSE120652) were collected for identification of differentially expressed genes [27,28] (https://www.ncbi.nlm.nih.gov/geo/query/acc.cgi?acc=gse120652 (accessed on 25 April 2021)). GSE14668 and GSE96851 were mRNA expression profiling datasets of hepatitis B virus (HBV)-related ALF. The unique acetaminophen-induced ALF data were found in GSE120652. GSE38941 was the dataset of HBV-ALF associated and chosen for validation [29]. Apart from GSE120652, which was from GPL6244, the other three datasets came from GPL570. All four datasets are liver-sample resourced. The specific accession information is presented in Supplementary Table S1.

### 2.2. Identification of Shared Ferroptosis-Related Differentially Expressed Genes (DEGs)

The Limma package in R software (4.0.3) was installed and utilized to analyze microarray data [30]. Logarithmic transformation was performed for the following calculations if needed and the expression calculation was processed. For patients who have four to five liver specimens in GSE14668 (HBV), GSE96851 (HBV), and GSE38941 (HBV), the averaged expressing value was calculated and used for further analysis. Adjusted *p* value < 0.05 and log FC > 1 was set as cut-off value. Taken the small amount of case and control size from GSE14668 (HBV) and GSE120652 (APAP) in account, we define *p* value instead of adjusted *p* value in these two datasets. Up-regulated and down-regulated DEGs were achieved, presenting by volcano plot in GraphPad Prism 8, a scientific tool to analyze, graph, and present data. A public ferroptosis regulator and marker genes database was retrieved from http://www.zhounan.org/ferrdb (accessed on 22 April 2021) [31]. The Venn diagrams were plotted via MyDraw software v4.3.0.

### 2.3. PPI Network Enrichment Analysis and Hub Genes Screening

Proteins encoded by shared ferroptosis-related DEGs were uploaded to Metascape (3.5), a gene annotation and analysis resource, for understanding the relationships between these ferroptosis proteins [32]. The STRING, BioGRID, OmniPath and InWeb_IM databases were integrated into Metascape for protein–protein interaction enrichment analysis [33,34,35]. MCODE was applied in Metascape to identify network modules specifically, and the functional description of the corresponding cluster was obtained by *p*-value [36]. The cytoHubba (0.1) plug-in of Cytoscape was used to screen out hub genes and hub factors [36].

### 2.4. Exploration of Regulators and Therapeutic Agents of Key Genes

In order to unravel the upstream pathway of key genes, the prediction of miRNAs that regulate the 10 hub genes were performed with the TargetScan (7.1) and miRTarBase (9.0) online tools, user-friendly public databases for microRNA biological targets prediction [37,38]. The corresponding lncRNA of overlapped miRNA performed by TargetScan and miRTarBase was targeted using DIANA-LncBase v2 via the experimental module. Prediction score > 0.95 was set as limited value for identifying potential lncRNA [39]. To discover potential drugs dealing with ALF, the identified 37 ferroptosis-associated DEGs were queried via The Drug Gene Interaction Database (DGIdb, https://dgidb.genome.wustl.edu (accessed on 10 October 2021)), L1000 fireworks display (L1000FWD, https://maayanlab.cloud/l1000fwd/ (accessed on 10 October 2021)), and Connectivity Map (cMAP, https://broad.mit.edu/cmap (accessed on 10 October 2021)) online tools. DGIdb stores over 100,000 drug-gene interactions or 42 potentially druggable gene categories. Users can retrieve all known or potential drugs through uploading a list of genes to the above website. L1000FWD is a visualized interactive network of over 16,000 drug- and small-molecule-induced transcriptional expression signatures. With the world’s largest perturbation-driven gene expression dataset, cMAP enables the exploration of relationships between diseases, cell physiology, and therapies. Usually, the assumed promising compounds were opposite to the transcriptional expression signatures. Venn diagrams were used to calculate the number of crosstalk compounds among three resources.

### 2.5. Validation of Ferroptosis-Associated Hub Genes

In order to validate whether the hub genes played inevitable roles in other mRNA expression profiling of ALF, a volcano plot of GSE38941 (HBV) was graphed. The heatmap of the expression of hub genes was constructed using GraphPad Prism 8.

### 2.6. Animals and ALF Model

Male mice from a C57BL/6j background (18–20 g, 7–8 weeks) were housed on a 12-h light-dark cycle and given a standard laboratory chow diet and water ad libitum. An ALF model was established in which mice were injected intraperitoneally with a single dose of 10 μg/kg lipopolysaccharide (LPS, Sigma-Aldrich, Darmstadt, Germany) and 450 mg/kg D-galactosamine (D-GalN, Sigma-Aldrich, Darmstadt, Germany). Niclosamide (40 mg/kg body weight, intraperitoneal) (Sigma-Aldrich, Darmstadt, Germany)/(5% DMSO + 2.5% Tween80 + 92.5% PBS) was pretreated 1 h earlier before LPS and D-GalN injection. Mice were sacrificed 5 h later after LPS/D-GalN injection and the liver tissues and blood samples were collected. Additional mice were observed closely to record survival rates at the endpoint.

Serum alanine aminotransferase (ALT) were measured by a fully automatic biochemical analyzer (AU5800, Beckman Coulter, Brea, CA, USA) in the Clinical Laboratory of Xiangya Hospital, Central South University. Hematoxylin and eosin (H&E, Beijing Solarbio Science & Technology Co., Ltd., Beijing, China) was used to stain liver paraffin sections. For the assessment of liver injury, Hepatic Injury Severity Scoring (HISS) was followed as previously reported [40]. In brief, capsular inflammation, steatosis, and ballooning degeneration was assigned a score of 0–3 and portal inflammation and spotty necrosis were attributed a score of 0–4 in the numerical grading system.

Animal research complied with relevant ethic regulations and was conducted following the care and use of laboratory animals established by Central South University.

### 2.7. Quantitative Real-Time Reverse-Transcription Polymerase Chain Reaction

Total RNA was extracted using TRIzol reagent (Invitrogen Life Technologies, Waltham, MA, USA). First-strand complementary DNAs were synthesized using a cDNA reverse transcription kit (CWBIO, Beijing, China). Quantitative real-time reverse-transcription polymerase chain reaction (qPCR) was performed using fluorescent SYBR Green I (CWBIO, Beijing, China) and respective primers (Supplementary Table S2).

### 2.8. Clinicopathological Information

Three confirmed patients with ALF admitted to the Department of Transplantation in Xiangya Hospital of Central South University were enrolled in our investigation. Another three age- and sex-matched patients with jaundice of unknown origin whose liver injury was mild were recruited as a control group. This study was approved by the Medical Ethics Committee of Xiangya Hospital of Central South University. The criterion for diagnosing ALF is as the definition in EASL clinical practice guidelines [41]. Briefly, ALF is characterized by a series of clinical symptoms including severe fatigue, obvious digestive manifestations, high bilirubin levels and poor coagulation, followed by encephalopathy. Liver samples of the control group were achieved by liver biopsy and ALF liver specimens came from pathological biopsy in the surgery of liver transplantation. All the liver samples were delivered to the Department of Pathology in Xiangya hospital and preserved in paraffin-embedded sections. Each group contains three liver samples. The detailed information is shown in Supplementary Table S3.

### 2.9. Immunohistochemistry

Immunohistochemistry was performed to investigate STMN1, 4-HNE, ACSL4, and STEAP3 expression in the liver tissues harvested from patients with ALF and jaundice of unknown origin as previously described [42]. In simple words, liver sections obtained from the Department of Pathology were deparaffinized with xylene and rehydrated with ethanol. After rinsing the tissue slides in distilled water, the liver slides were heated in sodium citrate buffer pH 6.0 at 95–100 °C for 20 min and cooled down at room temperature for antigen retrieval. After incubation of 3% hydrogen peroxide for 25 min and repeating washing with PBS, the slides were blocked with 3% bovine serum albumin for 30 min. Then the liver slides were incubated overnight at 4 °C with STMN1 antibody (Abcam, Cambridge, UK, ab52630)/4-HNE (Bioss, Boston, MA, USA, bs-6313R)/ACSL4 (ABclonal, ABclonal Technology, Wuhan, China, A6826)/STEAP3 (Bioss, Boston, MA, USA, bs-12825R) antibody solution covering them. Further, the liver sample area was incubated with secondary antibody solution for 50 min at room temperature, immersing in PBS buffer before and after the incubation. The sections were then stained with diaminobenzidine and stopped with distilled water rinsing under close monitoring to reach the ideal condition. Eventually, the slides were dehydrated with ethanol and xylene, followed by applying coverslips for further observation.

During the quantitative analysis for immunohistochemistry, the IHC Profiler plugin of Image J was used to value percentage of positive area and staining intensity automatically. High positive, positive, low positive and negative were graded into 3+, 2+, 1+ and 0. The final histochemistry score (H-score) ((percentage contribution of low positive × 1) + (percentage contribution of positive × 2) + (percentage contribution of high positive × 3)) was calculated in each section as previously reported [43].

### 2.10. Iron Content Assay

Liver tissue iron content was measured with the iron assay kit (Jiancheng Bio, Nanjing, China) according to the instructions. In brief, liver tissue was homogenized completely in saline, and supernatant after centrifuge was collected for the following reaction. Iron chromogenic agent and supernatant were mixed, and the OD value was measured to calculate iron concentration.

### 2.11. Target Prediction and Molecular Docking Analysis

The Canonical SMILES of niclosamide were uploaded to SwissTargetPrediction for target prediction. The targets were screened using “probability” greater than 0 as the criterion. Molecular docking was performed to explore the interaction between niclosamide and STAT3. The 3D structure of niclosamide in SDF format from PubChem data was imported into ChemBio3D Ultra 14.0 for energy minimization. The optimized small molecule was imported into AutoDockTools 1.5.6 for hydrogenation, charge calculation, charge assignment, and rotatable key setting. STAT3 (PDB ID: 6NJS) downloaded from the PDB database and PyMOL 2.3.0 were used to remove protein crystalline water, original ligands, etc. The protein structures were imported into AutoDockTools (v1.5.6). The protein binding sites were predicted using POCASA 1.1 and docked using AutoDock Vina 1.1.2.

### 2.12. Statistical Analysis

Data of clinical information were presented as means ± SEM. Statistical difference analysis between two groups was performed via 2-tailed unpaired Student’s *t*-test. *p* < 0.05 was considered statistically significant. Survival rates were calculated using the Kaplan–Meier method.

## 3. Results

### 3.1. 37 Ferroptosis-Related Genes Identified in ALF Datasets

**Figure 1:**
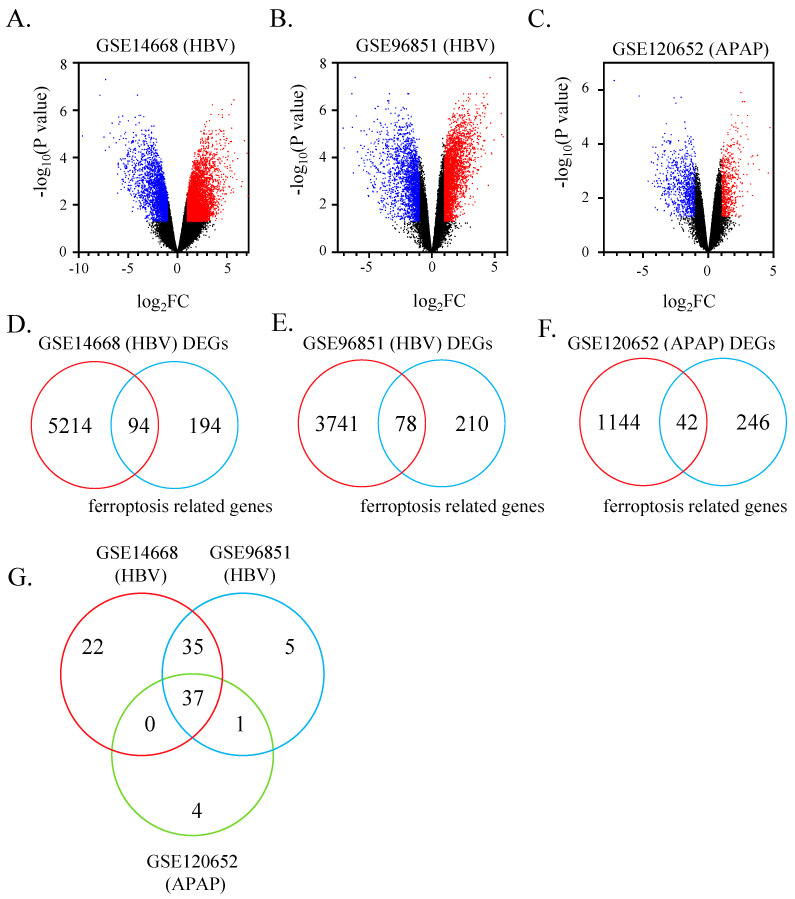
Identification of key ferroptosis genes in GEO datasets of ALF. (**A**–**C**) Volcano plot of GSE14668 (HBV), GSE96851 (HBV) and GSE120652 (APAP). Blue/red color represents down/up-regulated DEGs. *p* < 0.05. (**D**–**F**) Venn diagram shows the number of DEGs, ferroptosis-related genes, and overlapped genes between DEGs of three separate GEO datasets and ferroptosis-related genes. DEG’s definition was adj. *p* < 0.05 and log FC ≥ 1 in GSE96851 (HBV). The cut-off criterion in GSE14668 (HBV) and GSE120652 (APAP) is *p* < 0.05 and log FC ≥ 1 based on small experiment number. (**G**) Number of shared ferroptosis-related DEGs among three mRNA expression profiling datasets, presented in Venn diagram form.

To examine whether ferroptosis-related genes were differentially expressed in the liver of patients with ALF, we extracted the hepatic mRNA expression profiles of ALF from the GEO database firstly. DEGs of each dataset were shown in a volcano plot (Figure 1A–C). A total of 3534 significantly up-regulated genes and 1774 down-regulated genes were identified in GSE14668 (HBV). Besides, we found 2284 distinctly elevated genes and 1535 decreased genes in GSE96851 (HBV). A total of 357 dramatically up-regulated genes and 829 down-regulated genes were found in GSE120652 (APAP). As shown in the Venn diagram, 94, 78 and 42 significantly expressed ferroptosis-associated genes were obtained from GSE14668 (HBV), GSE96851 (HBV), and GSE120652 (APAP) separately, combing with ferroptosis-related genes from the public ferroptosis database (Figure 1D–F). Supplementary Table S4 shows the specific ferroptosis-associated DEGs in each accession. Subsequently, the Venn diagram presents overlapped genes among three groups (Figure 1G). From the perspective of comparison between two groups, the majority of ferroptosis-related genes are shared between two HBV ALF datasets, GSE14668 (HBV) and GSE96851 (HBV), which takes up to 92.3% of ferroptosis-related DEGs in GSE96851 (HBV) group. Interestingly, almost all hepatic ferroptosis-related DEGs of acetaminophen-induced ALF are included in two HBV-ALF groups, occupying 88.1% of ferroptosis-related genes in GSE120652 (APAP). Indeed, a total of 37 ferroptosis-related genes are shared among HBV-ALF and APAP-ALF microarray data. These 37 genes are in the same stages of increase/decrease in both the experiment and control groups.

### 3.2. PPI Network Establishment and Hub Factors Screening

**Figure 2:**
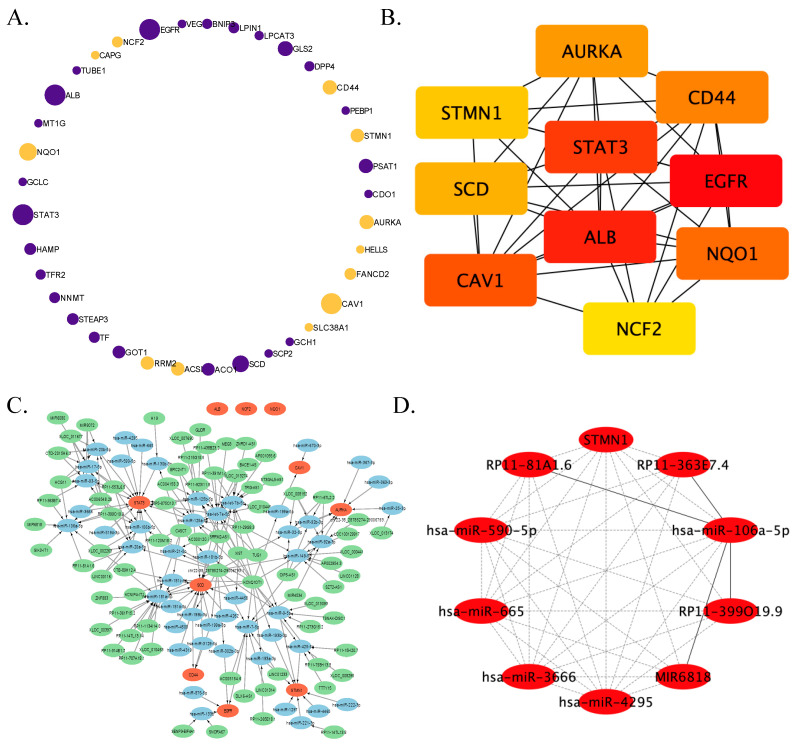
Identification of ferroptosis hub factors in ALF mRNA expression profiling datasets. (**A**) PPI network enrichment analysis was performed by Metascape. Each purple node represents a protein encoded by down-regulated ferroptosis-associated DEGs and yellow means a protein coded by up-regulated ferroptosis DEGs in patients with ALF. The more relationships with other proteins, the bigger the node. (**B**) Top ten hub genes identified by the cytoHubba Cytoscape plugin from 37 ferroptosis DEGs in three GEO datasets. The redder color means higher ranking by the MCC algorithm. (**C**) Predicted regulator network of hub genes. Red/blue/green color represents mRNA/miRNA/lncRNA. (**D**) Top ten factors identified by cytoHubba with DMNC, MNC, and clustering coefficient method.

In order to understand the relationship between these shared ferroptosis-associated DEGs discovered in the liver samples of patients with ALF, a PPI network was established (Figure 2A). In total, 12 ferroptosis-related DEGs increased in ALF; nevertheless, 25 genes dramatically decreased relative to the control group. Mainly synthesized in human liver and an important marker of liver function, albumin (ALB) was privileged with the most extensive relationship between other proteins encoded by ferroptotic key genes. Among these key genes, the top 10 hub genes were identified. These hub genes are as follows according to their degree calculated by the MCC method: ALB, epidermal growth factor receptor (EGFR), STAT3, caveolin 1 (CAV1), NAD(P)H quinone dehydrogenase 1 (NQO1), stearoyl-CoA desaturase (SCD), cluster of differentiation-44 (CD44), aurora kinase A (AURKA), stathmin (STMN1), and neutrophil cytosolic factor 2 (NCF2) (Figure 2B). 

For the guidance of future research, the mechanism of ferroptosis-related DEGs affecting the biological and pathological process of ALF is ready to be explored. With the help of integrated bioinformatics, the lncRNA-miRNA-mRNA regulatory network of hub genes could be predicted (Figure 2C). ALB, NQO1, and NCF2 were excluded from this network because they were estimated to be regulated by poorly conserved miRNA. This network consists of 77 lncRNAs, 32 miRNAs and 7 mRNAs. Two miRNAs, miR-181a-5p and let-7e-5p, had the most interacted lncRNAs, and STAT3 was regulated by the greatest number of miRNAs in the network. Notably, when the prediction score was limited to 1, the most popular lncRNA-chr22-38_28785274-29006793.1 was predicted to directly target almost all miRNAs. With the application of the cytoHubba Cytoscape plugin, ten hub factors of this network were screened out. Interestingly, STMN1 was the unique mRNA of hub factors, calculating by DMNC, MNC and clustering coefficient method (Figure 2D). Other hub RNAs are RP11-363E7.4, RP11-399O19.9, MIR6818, RP11-81A1.6, hsa-miR-106a-5p, hsa-miR-4925, hsa-miR-3666, hsa-miR-665, and hsa-miR-590-5p. 

### 3.3. Validation of Hub Genes and Role of Ferroptosis in ALF

**Figure 3:**
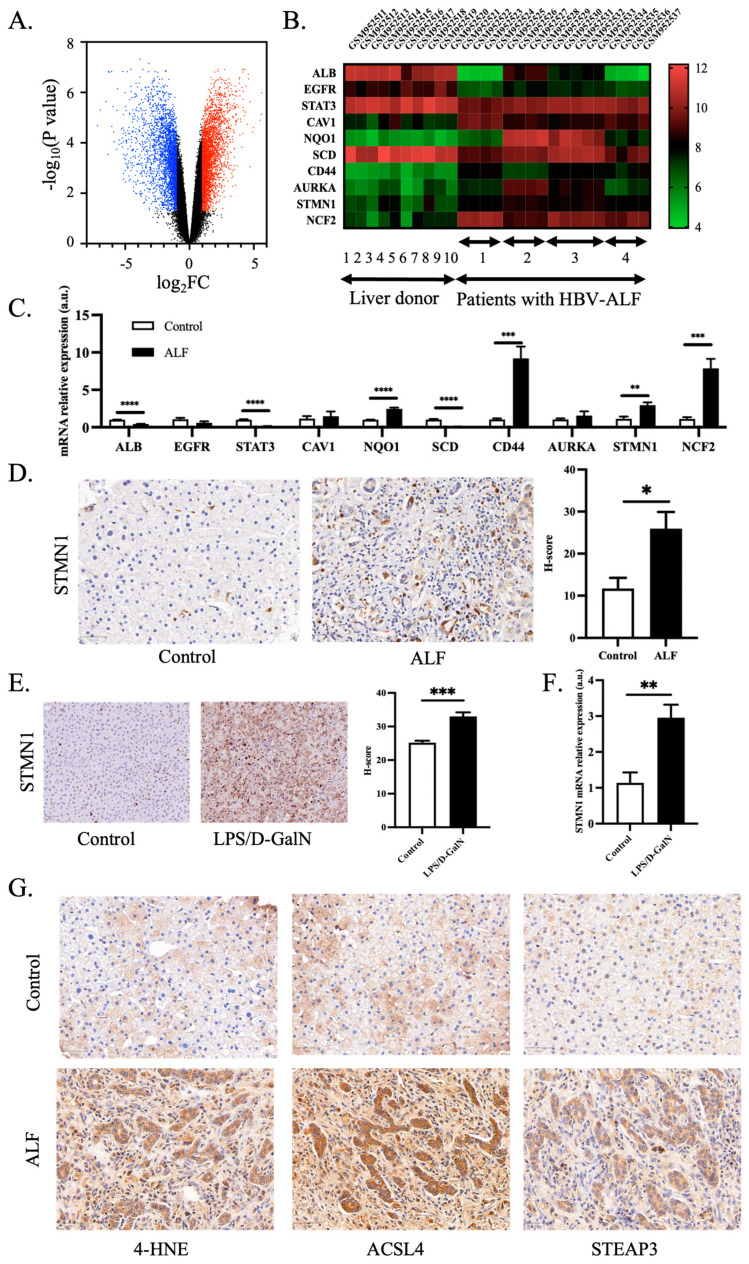
Validation of hub genes of ferroptosis-related DEGs in ALF. (**A**) Volcano plot of HBV-ALF dataset-GSE38941 (HBV). Up/down-regulated DEGs are shown in red/blue. *p* < 0.05. (**B**) Heatmap of expression of ten hub genes in validation dataset GSE38941 (HBV). Higher expression is shown in redder color and lower expression in greener color. (**C**) Liver gene expression of hub genes in mice model of LPS/D-GalN (normalized to GAPDH gene expression levels, *n* = 5 per group). (**D**) Liver paraffin section of control group and patients with ALF were stained with STMN1 antibody. Positive cells are stained brown. H-score was used to quantify positive area and intensity (*n* = 3 per group). (**E**) Liver paraffin sections of mice model of ALF were stained with STMN1 antibody. H-score was used to quantify positive area and intensity (*n* = 5 per group). (**F**) Liver gene expression of STMN1 5 h after LPS/D-GalN or vehicle treatment in mice model (normalized to GAPDH gene expression levels, *n* = 5 per group). (**G**) Representative pictures of immunohistochemistry in liver paraffin section of the control group and patients with ALF were stained with 4-HNE/ACSL4/STEAP3 antibody. 4-HNE/ACSL4/STEAP3 positive cells are stained brown. Bar = 50 μm. * *p* < 0.05, ** *p* < 0.01, *** *p* < 0.001, **** *p* < 0.0001.

To further validate ferroptosis-related hub genes in ALF, we examined the mRNA expression profiling in another GEO dataset of ALF. A total of 1743 genes were significantly down-regulated in liver samples of ALF in GSE38941 (HBV), and 2202 genes were robustly up-regulated in patients with ALF relative to the control group (Figure 3A). Encouragingly, these ten hub genes exhibit significant differences between the two groups and show the same trend as the previous three GEO datasets when comparing ALF and the control group (Figure 3B). Additionally, we performed validation of hub genes in the mice model. The results showed that the trends of all hub genes were the same as the validated GEO database, and seven of the hub genes were significantly different between the control group and those with LPS/D-GalN-induced ALF (Figure 3C). 

Given that STMN1 is one of the hub genes scarcely reported in the investigation of ALF and emerged out of the lncRNA-miRNA-mRNA regulatory network, we identified STMN1 as an important biomarker of ALF. We further validated the protein level of STMN1 in the control group and patients with ALF induced by HBV infection and the herbal medicine called Dingkun Dan. As shown in Supplementary Table S3, excluding the difference of sex and age, total bilirubin and alanine transaminase were substantially higher in the ALF group. Conversely, the prothrombin time activity was much lower relative to the control group. Obviously, the expression of STMN1 was expressed dramatically higher in the liver tissue of ALF compared to control liver tissue (Figure 3D). Furthermore, STMN1 protein and mRNA level were significantly elevated in the ALF model of animals (Figure 3E,F). In summary, STMN1 could serve as a new biomarker of ALF.

To further validate the role of ferroptosis in ALF of human subjects, we detected the protein level of lipid peroxidation marker 4-HNE and ferroptosis markers ACSL4 and STEAP3 in the livers of the control group and patients with ALF. As shown in Figure 3G and Supplementary Figure S1, 4-HNE, ACSL4 and STEAP3 were significantly increased in the ALF group. Overall, ferroptosis plays a vital role in ALF.

### 3.4. Identifying Promising Curative Compounds for ALF

**Figure 4:**
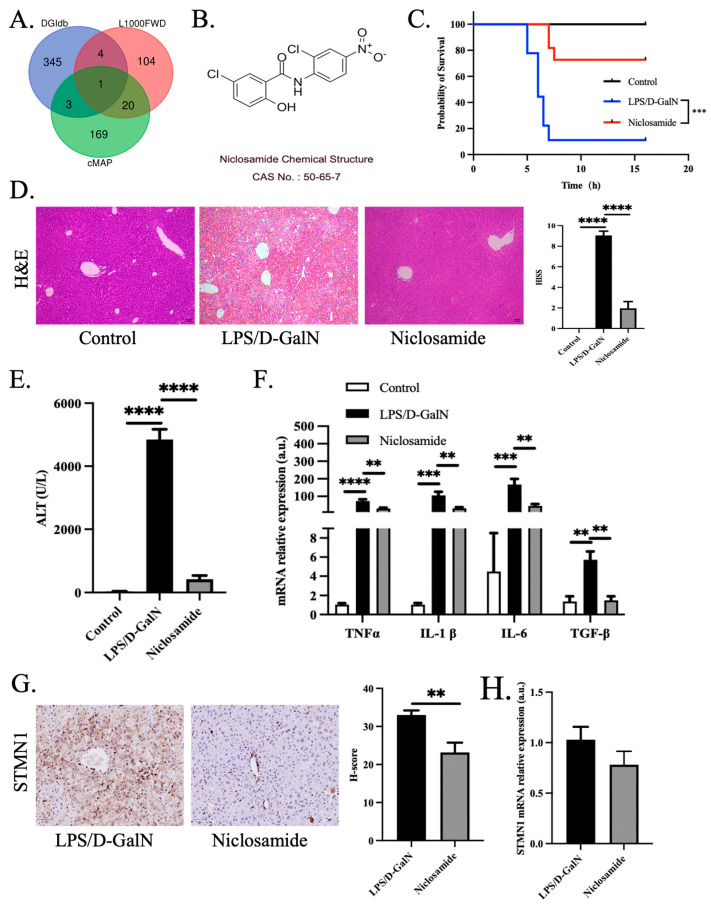
Niclosamide protected mice against LPS/D-GalN-induced ALF. (**A**) Prediction of overlapped drugs among three compounds databases: DGIdb, L1000FWD and cMAP (shown in blue/red/green circle), shown in Venn diagram form. Similarity scores ≤ −0.2 was set as cut-off value in L1000FWD. The small molecule compounds predicted by cMAP were narrowed down by limitation of cMAP score ≤ −0.66. (**B**) Chemical structure of niclosamide. (**C**) Survival rates following LPS (10 μg/kg body weight)/D-GalN (450 mg/kg body weight), pretreatment with niclosamide (40 mg/kg) or rescue with niclosamide (10 mg/kg). Control group, *n* = 5, LPS/D-GalN group, *n* = 9, niclosamide treatment group, *n* = 11. (**D**) Liver paraffin sections were stained with H&E agents. Control group, *n* = 5, LPS/D-GalN group, *n* = 5, niclosamide treatment group, *n* = 5. (**E**) Serum ALT levels. Control group, *n* = 5, LPS/D-GalN group, *n* = 7, niclosamide treatment group, *n* = 8. (**F**) Liver gene expression of inflammatory genes in mice model of LPS/D-GalN (normalized to GAPDH gene expression levels, *n* = 5 per group). (**G**) Liver paraffin sections of mice model of ALF were stained with STMN1 antibody (*n* = 5 per group). (**H**) Liver gene expression of STMN1 5 h later after LPS/D-GalN treatment in the absence or presence of niclosamide in mice model (*n* = 5 per group). Bar = 50 μm. ** *p* < 0.01, *** *p* < 0.001, **** *p* < 0.0001.

Since the hub genes had been validated, we next sought to discover promising curative drugs beneficial to ALF from the perspective of ferroptosis based on the 37 key genes. Through data mining from three compounds prediction resources, DGIdb, L1000FWD, and cMAP, several potential drugs were identified based on the signature of key genes (Figure 4A). Overlapping small molecule compounds were shown in detail in Table 1. It is worth discussing that niclosamide is the only drug predicted to be helpful to ALF covered by all three databases (Figure 4B). Niclosamide is a traditional anthelmintic drug, which is rarely absorbed after oral administration, and is excreted in the urine and feces [44]. Hepatic enzymes, along with enzymes found in cestodes and nematodes, have the ability to convert niclosamide into its corresponding amino derivatives [45]. Occasionally, niclosamide may give rise to allergies and gastrointestinal disorders, but it is not known to be associated with mutagenicity [44,46,47]. The top three overlapped compounds from L1000FWD and cMAP are BRD-K73610817, BRD-K08307026 and BRD-K81795824. 

To verify whether niclosamide could protect against ALF as predicted, we constructed an ALF mice model. In LPS/D-GalN-induced liver failure, almost all mice pretreated with niclosamide survived within our observation period, and their survival rate was substantially higher than the ALF group (Figure 4C). Moreover, liver injury (H&E) and serum ALT levels were distinctly decreased in the niclosamide pretreatment group (Figure 4D,E). Meanwhile, the expression of pro-inflammatory factors TNFα, IL-1β, IL-6, and TGF-β gene were significantly increased in the ALF group, and niclosamide significantly decreased the transcription level of inflammatory factors (Figure 4F). Besides, we detected STMN1 expression, the identified biomarker of ALF, with niclosamide intervention in LPS/D-GalN model. Niclosamide treatment downregulated the expression of STMN1 protein and mRNA level (Figure 4G,H). 

### 3.5. Niclosamide Protected against ALF via Ferroptosis

**Figure 5:**
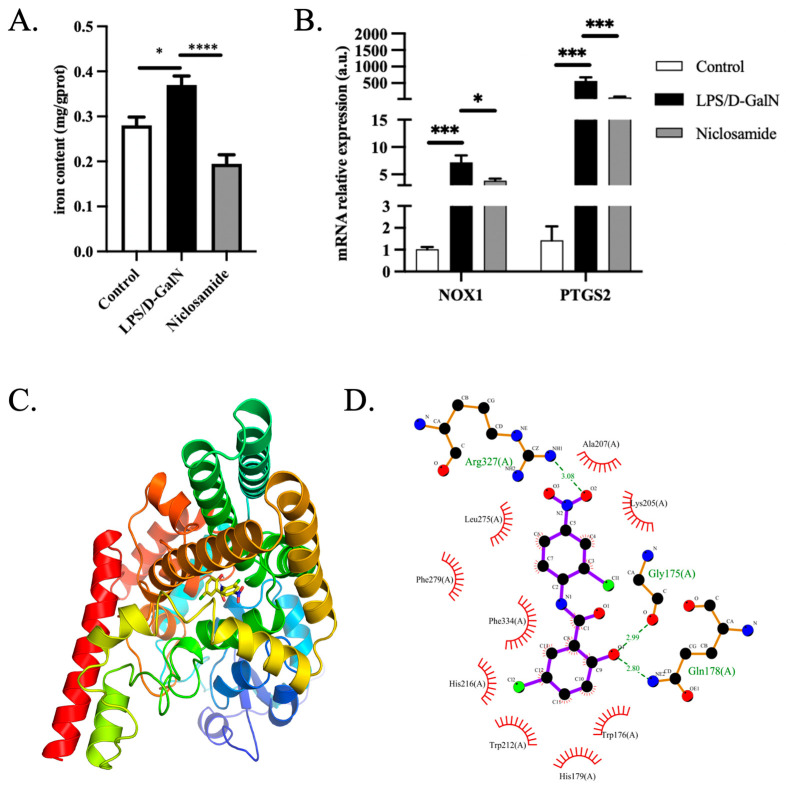
Ferroptosis was involved in the protective role of niclosamide in ALF. (**A**) Liver iron content. Control group, *n* = 7, LPS/D-GalN group, *n* = 9, niclosamide treatment group, *n* = 9. (**B**) Liver gene expression of NOX1 and PTGS2 (*n* = 5 per group). (**C**) Niclosamide was predicted to interact with STAT3. **(D)** Molecular docking in detail. * *p* < 0.05, *** *p* < 0.001, **** *p* < 0.0001.

Since niclosamide was identified by ferroptosis-related DEGs in ALF, there was a great chance that ferroptosis was involved in the protective role of niclosamide against ALF. To explore the potential mechanism of niclosamide in ALF, we examined the iron content and gene expression of ferroptosis classical markers. Compared to the LPS/D-GalN-induced ALF group, the liver iron levels of mice with niclosamide treatment were significantly lower (Figure 5A). Ferroptosis marker genes NOX1 and PTGS2 were significantly upregulated in ALF compared with the control group while the NOX1 and PTGS2 mRNA expression levels were significantly decreased in the niclosamide-treated group (Figure 5B). To predict the possible interaction target protein with niclosamide, SwissTargetPrediction was used. The protein encoded by the hub gene STAT3 was predicted to interact with niclosamide with the highest probability (Supplementary Table S5). Molecular docking analysis showed that the binding energy of niclosamide with STAT3 was −7.0 kcal/mol, which proved to be a good binding target (Figure 5C). Niclosamide interacted with STAT3 mainly through the formation of hydrogen bonds as well as hydrophobic forces (Figure 5D). The hydrogen bonds with Asp566 and Asn567 are 2.92 Å and 3.20 Å, respectively; hydrophobic forces interacted with Pro471, Asp570 and Ile569. Collectively, ALF may promote the activation of ferroptosis, while niclosamide administration significantly inhibited ferroptosis in ALF by interacting with STAT3. 

## 4. Discussion

In this investigation, we explored the role of ferroptosis in the progression of ALF, and predicted and validated niclosamide as a promising drug towards ALF. This is the first study of ferroptosis in patients with ALF with multiple-dimensional analysis ranging from upstream regulators to downstream pathways identified in silico. The study findings reveal that ferroptosis is an important pathway in ALF and broadens the future research direction in ALF.

In this investigation, we have validated that ferroptosis was activated in the final stage of ALF from human subjects of both HBV- and drug-induced ALF conditions, and an animal model of drug-induced liver failure. Lipid peroxidation drives ferroptosis and one of the important lipid peroxidation products is 4-HNE [48]. ACSL4 has been thought to be the important enzyme in ferroptosis [49]. Liver ablation of ACSL4 inhibited lipid peroxidation and APAP-induced hepatotoxicity [15]. STEAP3, PTGS2, and NOX1 are important ferroptosis evaluation markers [50,51,52]. We have found that lipid peroxidation marker 4-HNE, ferroptosis marker ACSL4, and STEAP3 protein were overexpressed in ALF patients, and PTGS2 and NOX1 mRNA expression combined with iron level increased in LPS/D-GalN-induced ALF. The upregulation of ferroptosis and the protective role of ferroptosis inhibitors in case of liver injury, established by cellular and murine models, have been widely accepted [53]. In the final stage of liver failure, ferroptosis inhibitors may become a promising treatment based on our in silico and in vivo results. Liver failure is considered to be stimulated by a complex pathophysiological mechanism wherein oxidative stress is thought to be a key factor [54]. One of the key biochemical characteristics of ferroptosis is iron accumulation. Iron overload leads to redox imbalance and cell death, and the predominant impaired organ is the liver [55]. Hepatocytes are the major iron storage sites and important sensors of iron levels, and iron overload causes progressive liver damage [56]. Dysregulation of the iron channel or exchange genes contributes to the iron metabolism disorder [11]. The iron channel genes were related to ALF in previous reports, e.g., inhibiting iron load via regulating the iron transporter protein DMT1/FPN1 axis could alleviate liver oxidative damage and protect hepatocytes from ferroptosis [57]. The NIK/IKKα/ROS/ferroptosis axis is involved in the progression of liver disease [18]. Ferroptosis is an essential pathophysiological process in the development of ALF. 

Construction of the lncRNA-miRNA-mRNA regulatory network helped identify the molecular signatures of ferroptosis regulation in ALF. LncRNA chr22-38_28785274-29006793.1—estimated to target almost all miRNAs—and two miRNAs—miR-181a-5p and let-7e-5p—predicted to interact with the most lncRNAs were identified in this study; these networks have rarely been reported in liver diseases. Hub nodes of ferroptosis-related regulatory networks comprising four lncRNAs, five miRNAs, and one mRNA were identified, indicating their importance in regulating ferroptosis and implying their role as potential biomarkers or drug targets in patients with ALF. Among these ten hub nodes identified, RP11-363E7.4, miR-106a-5p, miR-3666, miR-665, miR-590-5p, and *STMN1* facilitate cell death, whereas miR-4295 inhibits apoptosis in human glioma cell lines [58,59,60,61,62,63,64]. miRNA-106a-5p, which is directly targeted by four other lncRNAs in the hub nodes, was sponged and downregulated in breast cancer cells, leading to inhibition of ferroptosis [65]. All the core lncRNAs and miRNAs have not yet been investigated in liver injury or failure.

Using a small molecule compound prediction database, we identified several compounds that could potentially treat ALF. Niclosamide, as the unique common predicted drug treating ALF, proved to reverse ALF in an animal model induced by LPS/D-GalN. Niclosamide is an FDA-approved oral anthelmintic drug that disrupts the mitochondrial function of parasites, which recently was found to play a broad therapeutic role against cancer, metabolic diseases, bacterial and viral infection, etc. [66]. Mitochondrial uncoupling is the crucial mechanism in niclosamide functioning, which contributes to the decrease in ROS production [66,67]. Mitochondria serve as a primary source of ROS within cells [68]. To counteract the detrimental effects of excessive ROS, endogenous proteins known as mitochondrial uncoupling proteins (UCPs) exhibit inherent antioxidative activity in diverse conditions [69,70,71]. UCP2 and UCP3 are known to be acutely activated by ROS, which subsequently directly modulate the glutathionylation status of these uncoupling proteins. This modulation leads to a decrease in ROS emission and involvement in cell signaling mechanisms [72]. Among the UCP family, UCP2 exhibits a wide distribution and has been shown to uncouple oxidative phosphorylation, resulting in concurrent reductions in ROS production [67]. Uncoupling proteins 2 (UCP2) was proved to protect LPS-induced acute kidney injury by ameliorating mitochondrial dysfunction and oxidative stress [73]. ROS and RNS cooperate to affect proteins, lipids, and DNA modifications, leading to liver dysfunction [18,74]. Liver oxidative stress is the prominent mechanism in ALF. Therefore, mitochondria uncoupling may be involved in attenuating ALF progression by decreasing ROS level, which needs more investigation. 

Ferroptosis is initiated by lipid peroxidation relying on ROS production and iron overload [7]. Whether ferroptosis mediates the process of niclosamide inhibiting ALF through ROS decrease is deserved to be further explored. Notably, ferroptotic markers PTGS2 and NOX1 mRNA and iron content were robustly decreased in the niclosamide group which indicated that ferroptosis was blunted with niclosamide treatment. One of the potential mechanisms of niclosamide protecting mice from ALF was speculated to be through ferroptosis. Moreover, niclosamide was proved to alleviate immune response by decreasing cytokines secreted by murine macrophage [75]. Niclosamide exhibited immunomodulatory actions in the context of LPS/D-GalN-induced liver injury. Niclosamide reduces the production of pro-inflammatory cytokines, such as TNF-α and IL-6, in the liver. This modulation of the inflammatory response contributes to the attenuation of liver injury. The dysregulation of immunological regulatory mechanisms in liver diseases can disrupt the liver’s tolerogenic nature and lead to immune-mediated liver injury [20]. However, therapeutic interventions such as niclosamide hold promise in protecting against liver injury by targeting ferroptosis and modulating the inflammatory response. Other possible mechanisms for the protective role of niclosamide may be associated with the suppression pathway of Wnt/beta-catenin, NF-kappaB, signal transducer and activator of transcription 3 and notch signals which was validated in targeting cancer and liver fibrosis [76,77]. The top three overlapping uncharacterized compounds—BRD-K73610817, BRD-K08307026, and BRD-K81795824—previously discovered by computational analysis are valuable molecules for researchers and commercial drug companies investigating ALF therapy. Niclosamide and other predicted compounds may be applied to other liver diseases in the near future.

In this study, we identified ten hub genes according to their degree of importance and high connectivity in the network. Indeed, most proteins encoded by the hub genes have been identified in multiple ALF-associated experiments. STAT3 is a vital nuclear transcriptional factor which mediates signal transduction of cytokines and growth hormones, and which is activated in phosphorylation [78]. The STAT3 pathway is involved in the progression of ALF and the ratio of p-STAT3 to STAT3 increased in liver injury [79,80]. Though some regulators were reported to mediate the function of STAT3, the upstream pathway of STAT3 is still not well characterized. Niclosamide inhibited multiple oncogenic pathways including STAT3 [81]. Here, we predicted the protein encoded by the ferroptosis-related gene STAT3 as the target of niclosamide, implying that niclosamide protects against ALF via ferroptosis through combing with STAT3. STMN1 has been reported to be an important cytosolic phosphoprotein that is upregulated in all types of cancers and is positively correlated with poor prognosis in hepatocellular carcinoma [82,83]. Monitoring *STMN1* expression may be beneficial for early intervention in patients with HBV cirrhosis [84]. The expression of STMN1 was inhibited by treatment with the ferroptosis-inducer erastin [85]. The mRNA and protein level of STMN1 increased in CCl4-induced liver failure [86]. However, the role of STMN1 in ALF has not yet been fully investigated. In our study, the mRNA level of STMN1 was upregulated in LPS/D-GalN-induced ALF. Therefore, it is quite possible that STMN1 increases in common drug-induced liver failure. STMN1 is expressed at a very low level in control liver tissue, while its expression is extremely high in liver tissue of ALF; thus, STMN1 could serve as a candidate biomarker to observe the extent of ferroptosis during ALF and monitor the efficacy of treatment in ALF as a potential therapeutic drug.

Our study has several limitations. First, the current study is restricted by the small number of cases and controls as well as the lack of extensive categories of etiologies. Moreover, in vitro and in vivo validation experiments with adequate sample numbers are needed in the future to conclusively establish the relationship between ferroptosis and ALF, and especially to identify the role of niclosamide and STMN1. Clinical trial regarding to the protective implication of niclosamide in ALF is encouraged by the promising experiment in vivo. 

To our best knowledge, this is the first study to identify potential drugs and a lncRNA-miRNA-mRNA regulatory network that target ferroptosis-related genes in ALF based on integrated bioinformatics in humans. Especially, we identified niclosamide to be a promising drug for ALF treatment. In conclusion, our study may help advance our understanding of the role of ferroptosis in ALF, and the constructed regulatory network and potential drug prediction may be promising for devising therapeutic interventions in patients with ALF.

## Figures and Tables

**Table 1:** Specific compounds predicted to have activity against acute liver failure via drug prediction website.

Platforms	DGIdb and L1000FWD	DGIdb and cMAP	L1000FWD and cMAP
Predicted overlapping compounds	crizotinib itraconazole geldanamycin niclosamide PHA-665752	sunitinib niclosamide regorafenib TAK-285	BRD-K73610817 BRD-K08307026 BRD-K81795824 DG-041 BRD-K39757396 thapsigargin cyclosporin-a BRD-K60870698 VU-0418939-2 KO-143 VU-0418933-1 selamectin niclosamide BRD-K92202821 BRD-K19166598 BRD-K95196255 niguldipine BRD-A24021119 BRD-K78385490 CGP-71683 BRD-A50388635

## Data Availability

The data that support the findings of this study are available from the corresponding author upon reasonable request.

## Supplementary Materials

The following supporting information can be downloaded at: https://www.mdpi.com/article/10.3390/pharmaceutics15071950/s1, Figure S1: H score of ferroptosis biomarker in control group and patients with ALF; Table S1: Accession information of four mRNA expression profiling datasets associated with ALF; Table S2: qPCR primers; Table S3: Clinical information of case and control group; Table S4: Specific ferroptosis regulator and marker genes ranked by log FC value in each GEO dataset; Table S5: SwissTargetPrediction for niclosamide.
