# Supplementary material for: Repurposing Niclosamide as a Therapeutic Drug against Acute Liver Failure by Suppressing Ferroptosis
Source: Pharmaceutics. 2023 Jul 14;15(7):1950. doi: 10.3390/pharmaceutics15071950 (PMC10383467; doi:10.3390/pharmaceutics15071950)
Supplement: Supplementary file 1 [file pharmaceutics-15-01950-s001.zip › pharmaceutics-2387830-supplementary.pdf]

pharmaceutics

Supplementary Material: Repurposing Niclosamide as a Therapeutic Drug for Acute Liver Failure

Xiao Zhong, Xue-Gong Fan and Ruochan Chen

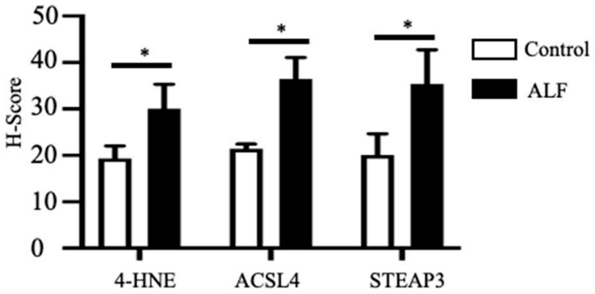

Supplementary Figure S1. H score of ferroptosis biomarker in control group and patients with ALF. Quantification of positive area and intensity in liver paraffin section stained with 4-HNE/ACSL4/STEAP3 antibody. (n=3 per group).

Supplementary Table S1. Accession information of four mRNA expression profiling datasets associated with ALF. GSE14668 (HBV), GSE96851 (HBV) and GSE120652 (APAP) were used for DEGs identification and GSE38941 (HBV) for validation.

Accession No.	Platform	Etiology	Sample size Case/Control	Organism	Experiment type	Country	Public year
GSE14668	GPL570	HBV	8/20	Homo sapiens	Expression profiling by array	USA	2010
GSE96851	GPL570	HBV	17/10	Homo sapiens	Expression profiling by array	Italy	2018
GSE120652	GPL6244	APAP	3/3	Homo sapiens	Expression profiling by array	USA	2020
GSE38941	GPL570	HBV	16/10	Homo sapiens	Expression profiling by array	USA	2012

Supplementary Table S2. qPCR primers.

Genes	Forward	Reverse
Gapdh	AGGTCGGTGTGAACGGATTTG	TGTAGACCATGTAGTTGAGGTCA
Tnfα	CATCTTCTCAAAATTCGAGTGACAA	TGGGAGTAGACAAGGTACAACCC
Il-1β	GCCTTGGGCCTCAAAGGAAAGAATC	GGAAGACACAGATTCCATGGTGAAG
Il-6	AGCCAGAGTCCTTCAGA	GGTCCTTAGCCACTCCT
Tgf-β	CTCCCGTGGCTTCTAGTGC	GCCTTAGTTTGGACAGGATCTG
Stmn1	GCCAGTCTCTTGTCTTCTGTT	CCTCCCGGTTCTCTTTGTTAG
Nox1	GCACAGCTTCCACACTTTC	CTCTCAGGTCGGCCAAG
Ptgs2	GTCATTGGTGGAGAGGTGTATC	CAGGAGGATGGAGTTGTTGTAG

Supplementary Table S3. Clinical information of case and control group (n=3 per group). Data are presented as mean ± SEM. *p<0.05, 2-tailed unpaired Student's t test.

Clinical parameters	ALF	Control
Sex (M/F)	2/1	2/1
Age (years)	40.3± 3.3	45.3± 4.1
Total bilirubin (umol/L)	463.9± 20.9*	28.2± 5.6
Alanine transaminase (U/L)	188.4± 10.1*	31.9± 4.5
Prothrombin time activity (%)	15.9± 1.3*	103.9± 2.5

Supplementary Table S4. Specific ferroptosis regulator and marker genes ranked by log [FC] value in each GEO dataset. Genes regarding ferroptosis retrieved from ferroptosis database.

**Accession**	**GSE14668** (HBV)				**GSE96851** (HBV)		**GSE120652** (APAP)	
Ferroptosis	**Up**		**Down**		**Up**	**Down**	**Up**	**Down**
related	RRM2	SRC	TFR2	CEBPG	RRM2	TFR2	STMN1	TFR2
DEGs	SLC7A11	TP63	CDO1	GCLC	NQO1	CDO1	CAPG	SCP2
	NQO1	ZFP69B	NNMT	LPCAT3	NCF2	ALB	HELLS	CDO1
	G6PD	MAP1LC3A	GLS2	GCH1	CAPG	HAMP	NCF2	PEBP1
	CAPG	JDP2	CXCL2	DRD4	SLC7A11	TF	FANCD2	MT1G
	HELLS	PML	ALB	TUBE1	CD44	GLS2	DDIT4	NNMT
	CD44	TGFBR1	SCD	EGFR	ALOX5	STEAP3	AURKA	TUBE1
	RB1	CYBB	HAMP	FH	TXNIP	NNMT	NQO1	EGFR
	TXNIP	STMN1	TF	SNX4	TLR4	CXCL2	ACSL4	STAT3
	NCF2	NFE2L2	HNF4A	TRIB3	ABCC1	HNF4A	RRM2	HAMP
	RGS4	GDF15	STEAP3	ZEB1	AURKA	CBS	CAV1	PSAT1
	TAZ	ACSL4	PLIN4	LPIN1	FANCD2	VEGFA	SLC38A1	PCK2
	FANCD2	HIC1	DPP4	ARNTL	RGS4	PSAT1	CD44	VEGFA
	SLC2A6	SQSTM1	VEGFA	KRAS	HMOX1	MT1G		DPP4
	ASNS	SLC7A5	PROM2	HSPA5	GDF15	BNIP3		SCD
	ATM	HMOX1	CBS	ACO1	HELLS	GOT1		STEAP3
	ALOX5	FTH1	BNIP3	STAT3	CYBB	SCP2		LPIN1
	TLR4	CDKN2A	MT1G	PCK2	G6PD	LPCAT3		BNIP3
	AURKA	HMGB1	TFRC	HSD17B11	ASNS	CEBPG		GLS2
	MYB	SLC38A1	SCP2	CISD2	CAV1	EGFR		LPCAT3
	MUC1	NF2	GPX2	PRDX6	SLC2A3	GCLC		TF
	ABCC1	ENPP2	PEBP1	ANO6	ENPP2	SCD		GCLC
	PGD	SLC3A2	PSAT1	ARRDC3	FTH1	GCH1		GOT1
	KLHL24	ULK2	PRKAA1	ALOX12B	SLC38A1	PEBP1		GCH1
	CAV1	ATG7	DUOX1	ELAVL1	STMN1	GPT2		GPT2
	SAT1	YWHAE	GOT1		JDP2	PRKAA1		ACO1
	JUN	CA9			MAPK1	TUBE1		ZFP36
	SLC2A3	MAPK3			ACSL4	SOCS1		ARRDC3
	FLT3	SRXN1			TSC22D3	DPP4		ALB
					KLHL24	STAT3		
					TGFBR1	GPX2		
					SLC2A6	TRIB3		
					MYB	LPIN1		
					ATM	HSPA5		
					SAT1	ARNTL		
					RB1	PLIN4		
					HMGB1	SNX4		
					PROM2	FH		
					PRKAA2	HSD17B11		
						ACO1		
						CISD2		

Supplementary Table S5. SwissTargetPrediction for niclosamide.

Target	Uniprot ID	ChEMBL ID	Probability*	Known actives (3D/2D)
STAT3	P40763	CHEMBL4026	1	1 / 2
KCNMA1	Q12791	CHEMBL4304	0.130791955	11 / 19
SAE1 UBA2	Q9UBE0 Q9UBT2	CHEMBL2095174	0.097874534	2 / 0
PIK3CG	P48736	CHEMBL3267	0.097874534	8 / 0
KDM4E	B2RXH2	CHEMBL1293226	0.097874534	2 / 0
KDM5C	P41229	CHEMBL2163176	0.097874534	1 / 0
KDM4A	O75164	CHEMBL5896	0.097874534	1 / 0
KDM4D	Q6B0I6	CHEMBL6138	0.097874534	1 / 0
PIK3CA	P42336	CHEMBL4005	0.097874534	5 / 0
PTGS1	P23219	CHEMBL221	0.097874534	4 / 3
PTGS2	P35354	CHEMBL230	0.097874534	6 / 2
PTK6	Q13882	CHEMBL4601	0.097874534	2 / 0
FADS1	O60427	CHEMBL5840	0.097874534	0 / 10
DHFR	P00374	CHEMBL202	0.097874534	2 / 0
DHODH	Q02127	CHEMBL1966	0.097874534	19 / 0
MAPK14	Q16539	CHEMBL260	0.097874534	5 / 7
CES2	O00748	CHEMBL3180	0.097874534	1 / 0
TTR	P02766	CHEMBL3194	0.097874534	4 / 0
CNOT7	Q9UIV1	CHEMBL3616361	0.097874534	4 / 0
ERCC5	P28715	CHEMBL4736	0.097874534	19 / 0
FEN1	P39748	CHEMBL5027	0.097874534	28 / 0
PDK1	Q15118	CHEMBL4766	0.097874534	0 / 96
PLAT	P00750	CHEMBL1873	0.097874534	0 / 21
F10	P00742	CHEMBL244	0.097874534	0 / 31
PLAU	P00749	CHEMBL3286	0.097874534	0 / 53
